# Fabrication of High Gas Barrier Epoxy Nanocomposites: An Approach Based on Layered Silicate Functionalized by a Compatible and Reactive Modifier of Epoxy-Diamine Adduct

**DOI:** 10.3390/molecules23051075

**Published:** 2018-05-03

**Authors:** Ran Wei, Xiaoqun Wang, Xu Zhang, Chen Chen, Shanyi Du

**Affiliations:** 1 School of Materials Science and Engineering, Beihang University, Xueyuan Road 37, Beijing 100191, China; bhweiran@163.com (R.W.); hippo_zhangxu@sina.com (X.Z.); sydu@hit.edu.cn (S.D.); 2 Shanghai Aircraft Manufacturing Co., Ltd., Shanghai 201324, China; ZY1501103@buaa.edu.cn

**Keywords:** epoxy-diamine adduct, layered silicate, nanocomposite, small-angle neutron scattering, gas barrier property

## Abstract

To solve the drawbacks of poor dispersion and weak interface in gas barrier nanocomposites, a novel epoxy-diamine adduct (DDA) was synthesized by reacting epoxy monomer DGEBA with curing agent D400 to functionalize montmorillonite (MMT), which could provide complete compatibility and reactivity with a DGEBA/D400 epoxy matrix. Thereafter, sodium type montmorillonite (Na-MMT) and organic-MMTs functionalized by DDA and polyether amines were incorporated with epoxy to manufacture nanocomposites. The effects of MMT functionalization on the morphology and gas barrier property of nanocomposites were evaluated. The results showed that DDA was successfully synthesized, terminating with epoxy and amine groups. By simulating the small-angle neutron scattering data with a sandwich structure model, the optimal dispersion/exfoliation of MMT was observed in a DDA-MMT/DGEBA nanocomposite with a mean radius of 751 Å, a layer thickness of 30.8 Å, and only two layers in each tactoid. Moreover, the DDA-MMT/DGEBA nanocomposite exhibited the best N_2_ barrier properties, which were about five times those of neat epoxy. Based on a modified Nielsen model, it was clarified that this excellent gas barrier property was due to the homogeneously dispersed lamellas with almost exfoliated structures. The improved morphology and barrier property confirmed the superiority of the adduct, which provides a general method for developing gas barrier nanocomposites.

## 1. Introduction

Due to the outstanding mechanical performance, adhesive property, processability, and low cost, epoxy resin has become one of the most widely used thermoset polymers for a wide range, of applications, including adhesives, coatings, storage containers for gas and liquid substances and as a matrix for composites. Some of these applications require not only excellent mechanical properties, but also superior gas barrier capabilities [1,2]. However, because of the multiplicity of the motion units and the creep property of polymer molecules, epoxy resin has the intrinsic drawback of inherent permeability to gases and vapors. Thus, their general applications in the aforementioned fields are limited [3,4]. Numerous attempts have been made to improve the gas barrier properties of epoxy resin, such as copolymerization or blending modification technology, multilayer composite technology, surface coating technology, and nano-filler modification technology [5,6,7,8,9]. Among them, the incorporation of layered nanomaterials with high aspect ratios, e.g., montmorillonite (MMT) [10,11] and graphene oxide (GO) [12,13], has been proved to be an efficient method, which could make the pathways of gas-penetrant molecules much longer and more tortuous, thus prolonging the diffusion time of gas molecules throughout the polymer. However, the bottlenecks which remain to be overcome are the poor dispersion of nano-layers, and weak interfacial interactions with polymeric resins due to the incompatibility between inorganic fillers and organic matrices [14].

Since the first attempt at preparing MMT/elastomer nanocomposites in 1950 [15], considerable work has already been undertaken on the functionalization of MMT to improve compatibility, in which, organic modifiers are intercalated into the interlayer via cation exchange process [16,17,18,19,20,21]. Among them, the most commonly used types were long-chain alkyl ammonium salt [16,17,18] and polyether ammonium salt [19,20,21]. Although these approaches of chemical functionalization were proved to be conductive to dispersion, a large gap still existed between the barrier properties of nanocomposites and theoretical results, owing to the limited compatibility between sheet and matrix [22]. In addition, since the interaction between fillers and matrix was just an adsorption process, the weak interfaces would also deteriorate the properties of nanocomposite [23].

In recent years, with the rapid development of graphene and graphene/polymer nanocomposites, the functionalization methods for nanomaterials are also promoted. Notably, grafting molecular chains of the polymer matrix onto nano-fillers is an efficient method to provide not only excellent compatibility, but also reactivity with matrix [24,25,26]. For instance, Wan et al. synthesized diglycidyl ether of bisphenol A (DGEBA) type epoxy composites filled with DGEBA functionalized GO (DGEBA-f-GO). The results showed a significant enhancement in compatibility and dispersion of DGEBA-f-GO sheet in DGEBA type epoxy matrix, resulting in a prominent enhancement in tensile property, interfacial interaction, and thermal stability in epoxy composites [26]. However, in the case of MMT, it could not be functionalized by epoxy molecules directly via either grafting or cation exchange methods. Therefore, a new kind of modifier, epoxy-monoamine adduct, was synthesized by reacting di-functional epoxy with monoamine (molar ratio of epoxy ring and active hydrogen was 1:1), resulting in better compatibility and thermal stability than commercial organic MMT modified by long-chain alkyl ammonium salts [27,28].

Moreover, since the improvement in gas barrier properties of epoxy nanocomposite is strongly dependent on the morphology of the layered fillers in the polymer matrix, including dispersion and exfoliation, the characterization method for nanomaterials is of critical importance. Due to the limitations of traditional characterizations like X-ray diffraction (XRD) and transmission electron microscopy (TEM), which could provide only local information on the surface of samples, the comprehensive morphology information of nanocomposites is still elusive, which has limited the development and application of these materials. Small-angle neutron scattering (SANS) is a powerful technique for studying the micro and nano-domain structures in nanocomposites, due to many unique features like high sensitivity, body probe and so forth [29]. Over the past decade, SANS has been widely used to probe the dispersion or exfoliated extent of layered nanomaterials in a solvent [30,31] or polymer matrix [32,33]. Most recently, it was further utilized to study the relationship between the morphology and properties of nanocomposites [32].

In the present study, to prepare a modifier with both compatibility and reactivity with epoxy matrix, a novel epoxy-diamine adduct (DGEBA-D400 adduct) was designed and synthesized by reacting epoxy monomer (DGEBA) with curing agent diamine (polyether amine D400) to functionalize MMT (DDA-MMT). Terminating with epoxy ring and amine groups, the adduct could not only functionalize MMT via cation exchange, but could also participate in the curing of DGEBA/D400 matrix via chemical bonding. Thereafter, Na-MMT, DDA-MMT and a kind of polyether amine modified MMT (T5000-D400-MMT) were incorporated into epoxy resin to manufacture nanocomposites. As reported in our previous work, T5000-D400-MMT was a co-intercalated MMT, functionalized by curing agent D400 and long-chain polyether amine T5000 successively, which possessed relatively good compatibility with matrices in addition to a large basal distance [22,34]. The relationship between surface functionalization, morphology and gas barrier property of the nanocomposites were systematically evaluated. Besides XRD and TEM, SANS was also utilized to characterize the morphology of MMTs and nanocomposites. The results showed that improved dispersion and exfoliation were observed in DDA-MMT/DGEBA nanocomposites compared to those reinforced by Na-MMT and T5000-D400-MMT. Moreover, the gas barrier properties were evaluated and compared with the modified Nielsen model to further understand the role of surface functionalization with the self-made compatible and reactive epoxy-diamine adduct. This research aimed at fabricating high gas barrier epoxy nanocomposite by incorporating layered silicate, which was functionalized by a compatible and reactive modifier of epoxy-diamine adduct.

## 2. Materials and Methods

### 2.1. Materials

Sodium type montmorillonite (Na-MMT) (cation exchange capacity 100 meq/100 g) was purchased from Zhejiang Fenghong Clay Chemicals Co., Ltd. (Huzhou, China). The diglycidyl ether of bisphenol A (DGEBA, epoxy value 0.51) type epoxy was supplied by Blue Star Petrochemical Co., Ltd. (Wuxi, China). The polyether amine series, including diamines (Jeffamine^®^D400) and triamine (Jeffamine^®^T5000), were obtained from Huntsman Corporation (Woodlands, TX, USA). Other chemicals employed in this research were purchased from Beijing Chemical Works (Beijing, China) and used directly.

### 2.2. Sample Preparation

#### 2.2.1. Synthesis of DGEBA-D400 Adduct (DDA)

With a molar ratio of 1:1, epoxy monomer DGEBA was blended with polyether diamine D400 by mechanical stirring and reacted at 55 °C for 4 h. Correspondingly, the molar ratio of epoxy groups and active hydrogen in amine groups was 1:2. Excessive amine was utilized to avoid cross-linking, and to obtain linear molecules.

#### 2.2.2. Functionalization of MMT

Prior to the functionalization of MMT, organic modifiers, including polyether amines, the epoxy-diamine adduct, and concentrated hydrochloric acid (HCl, 37 wt %) were dissolved in isovolumetric acetone. Then, the solutions were mixed and stirred for 3 h in an ice-water bath to obtain amine hydrochloride. The molar ratio of amines and hydrogen ions was stoichiometrically equivalent.

For mono-intercalated MMT including DDA-MMT and D400-MMT, the preparation procedure was as follows. First, Na-MMT (30 g) was dispersed in deionized water (1500 mL) and stirred for 72 h. Second, the amine hydrochloride solution was slowly added to the suspension to reach 1.5 times of the exchange capacity of Na-MMT, and stirred for 4 h at 50 °C. Then the suspension was centrifuged (3000 rad/min, 30 min) and washed twice with acetone to remove the unreacted amine hydrochloride.

For co-intercalated MMT of T5000-D400-MMT, T5000 hydrochloride solution was added dropwise to the D400-MMT suspension. After 4 h of stirring at 50 °C, the resulting co-intercalated MMT suspension was centrifuged and washed twice with acetone to remove unreacted amine hydrochloride.

#### 2.2.3. Manufacture of MMT/DGEBA Nanocomposite

First, functionalized MMTs were mixed with epoxy monomer DGEBA by mechanical agitating for 30 h to swell sufficiently, and further processed by ball-milling for 60 h. The high shear stress generated from milling impact could promote the dispersion and exfoliation of MMT layers. Second, curing agent D400 was added and mixed up by mechanical stirring. Afterwards, the mixture was poured into steel molds and cured at 70 °C for 2 h, 110 °C for 2 h and 150 °C for 3 h. To remove the trapped air, the mixture was degassed at 0.1 MPa for 30 min prior to curing. The weight ratio of DGEBA and curing agent D400 was 100:59. The clay content was adjusted to 3 wt % of the weight of the resin matrix. The specific processes are presented in Table 1.

### 2.3. Characterization of Materials

#### 2.3.1. Characterization

Gel permeation chromatography (GPC) measurement was conducted using a Waters 515-717-2410 (Waters, Milford, CT, USA) GPC system with a refractive-index (RI) detector. Samples were dissolved in THF (1 mg·mL^−1^) and injected by Waters 717 autosampler (0.6 mL·min^−1^).

A Fourier transform infrared (FTIR) measurement was carried out using a Nicolet Nexus 470 FTIR spectrometer (Thermo Nicolet Corporation, Waltham, MA, USA). The wavenumber range was from 4000 cm^−1^ to 750 cm^−1^.

Differential scanning calorimetry (DSC) and thermogravimetric (TG) analyses were conducted using a thermo-analyzer STA 449 C (Netzsch Group, Bavarian State, Germany) at a heating rate of 10 °C/min from 30 to 900 °C in a nitrogen atmosphere.

Transmission electron microscopy (TEM) was utilized to detect the morphology of MMTs in the epoxy resin matrix using a JEM-2100F (JEOL Ltd., Tokyo, Japan) under an acceleration voltage of 100 kV. All nanocomposites were collected on formvar-coated copper grids after being cut into ultrathin specimens (with thickness of about 100 nm).

The X-ray diffraction (XRD) patterns of MMTs were collected using a D/Max2200pc X-ray diffractometer (Rigaku, Tokyo, Japan) with Ni-filtered Cu Kα radiation (40 kV, 40 mA). The scan angles ranged from 3° to 15° (2θ), and the scan rate was 2° (2θ)/min. The small-angle X-ray scattering (SAXS) experiments were performed at D/Max2500pc X-ray diffractometer (Rigaku, Tokyo, Japan) with Ni-filtered Cu Kα radiation (18 kV, 40 mA). The scan angles ranged from 0.6° to 10° (2θ), with a scan rate of 1° (2θ)/min. Generally, basal spacing d_(001)_ of MMT can be calculated from the first-order diffraction peak (where n is 1) via Bragg’s law.

The small-angle neutron scattering (SANS) experiments were performed on the BILBY instrument at Australian nuclear science and technology organization (ANSTO, Sydney, NSW, Australia). Samples were measured in 2 mm quartz Hellma cells and equilibrated at 25 °C. Neutrons with a range of wavelengths from 4 to 13 Å (wavelength resolution is 10%) were used to cover a q-range from 0.002 to 0.992 Å^−1^. After being reduced using Mantid software and solvent subtracted, the data were presented as plots of the absolute intensity I(q) versus the wave vector q (Equation (1)), where θ_2_ is the scattering angle and λ_2_ is the wavelength of neutrons.

(1) $$q=\frac{4\pi \sin\theta_{2}}{\lambda_{2}}$$

#### 2.3.2. Gas Permeability Test

The through-the-thickness gas permeability of MMT/epoxy nanocomposites was measured by VAC-V1 gas permeability tester (Labthink Instruments Co., Ltd., Jinan, China) according to ASTM D 1434-82 (2015), which covers the standard test method for determining the gas permeability of plastic films. The specific test procedure is as follows. Prior to testing, one specimen was mounted in the gas diffusion cell so as to form a sealed barrier between the two chambers, named high-pressure chamber and low-pressure chamber. After being evacuated to vacuum, a flow of N_2_ at a pressure of 0.1 MPa was introduced to the high-pressure chamber, and the permeating gas was received by the low-pressure chamber. By monitoring the pressure changes in the low-pressure chamber over time, the gas transmission rate (Q_g_, cm^3^·m^−2^∙(24 h)^−1^∙Pa^−1^) and transmission coefficient (p_g_, cm^3^∙cm·cm^−2^∙s^−1^∙Pa^−1^) could be calculated based on Equations (2) and (3):

(2) $$Q_{g}=\frac{\Delta p}{\Delta t}\times \frac{V}{S_{1}}\times \frac{T^{\prime }}{{P^{\prime }T}_{1}}\times \frac{24}{\left(p_{1}-p_{2}\right)}$$

(3) $$p_{g}=\frac{\Delta p}{\Delta t}\times \frac{V}{S_{1}}\times \frac{T^{\prime }}{{P^{\prime }T}_{1}}\times \frac{D_{1}}{\left(p_{1}-p_{2}\right)}=1.1574\times 10^{-9}Q_{g}\times D_{1}$$

where ∆p/∆t is the gas pressure change per unit time (Pa·h^−1^) in the low-pressure chamber in steady-state permeation, while V, S_1_, T_1_, D_1_ and p_1_ − p_2_ stand for volume of low-pressure chamber (cm^3^), test area (m^2^), test temperature (K), and thickness (cm) of the specimen and the pressure difference on both sides of the sample. T’ and P’ represent respectively the temperature (273.15 K) and pressure (1.0133 × 10^5^ Pa) at the standard thermodynamic state. In this research, V is 6.12 cm^3^, S_1_ is 38.48 cm^2^, and T_1_ is 25 °C. The samples were wafers with a diameter of 97 mm and a thickness of approximately 1 mm. Three samples were tested to obtain average values.

## 3. Results and Discussion

### 3.1. Characterization of DGEBA-D400 Adduct (DDA) and DDA-MMT

**Figure 1:**
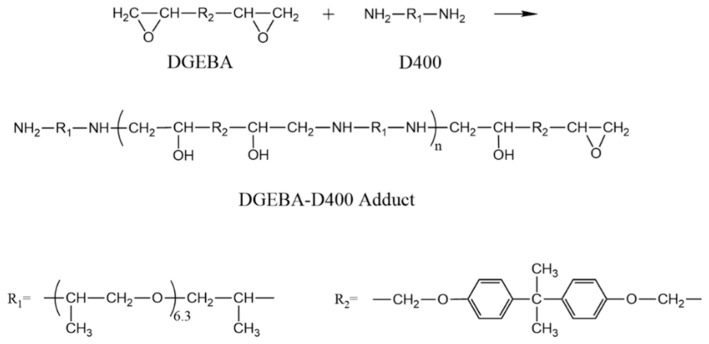
Synthesis of DGEBA-D400 adduct by nucleophilic addition reaction between DGEBA and D400.

First, DGEBA-D400 adduct (DDA) was synthesized using a nucleophilic addition reaction between DGEBA and curing agent D400. To avoid crosslinking during the preparation process, the reaction conditions were determined by the DSC curve and the viscosity growth rate of DGEBA/D400 under heating, which could ensure that the molecular chains grow slowly and stop before crosslinking. The chemical reaction is depicted in Figure 1.

In order to investigate the molecular composition and structural features of DDA and the corresponding modified MMT, characterizations of GPC, FTIR and SAXS/XRD were carried out. The molecular weights and distribution of DDA investigated by GPC are shown in Table 2. As can be observed, the adduct consisted of two components. The presence of the main component with a number-average molecular weight (M_n_) of 1460 g/mol confirmed the successful reaction between DGEBA and D400. The other component possessed an M_n_ of 368, which was comparable to that of the DGEBA monomer, suggesting that little DGEBA retained in the resulting adduct. As it could not be acidified, the small amount of residual DGEBA would be removed during the subsequent acidification process.

**Figure 2:**
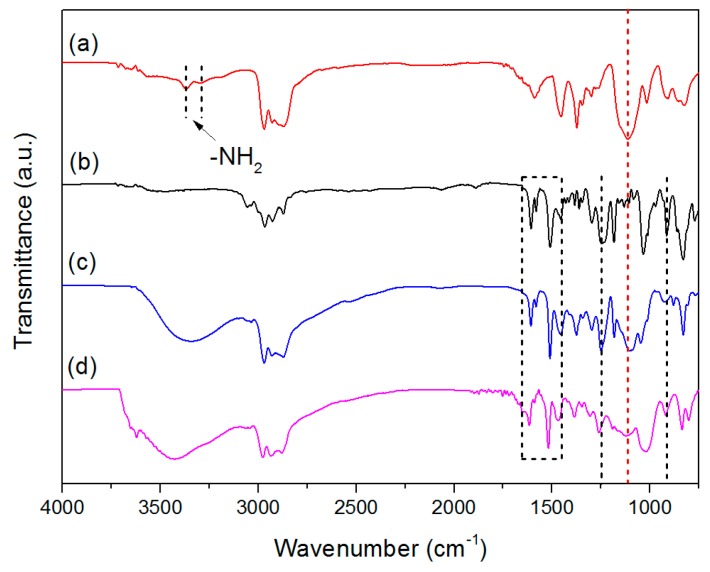
FTIR spectra of D400 (a) DGEBA (b), DGEBA-D400 adduct (c) and DDA-MMT (d).

FTIR spectra of DGEBA, D400, DDA and DDA-MMT are presented in Figure 2. The characteristic absorptions of 1,4-substituted benzene (3055 cm^−1^, 1607 cm^−1^, 1581 cm^−1^ and 1510 cm^−1^) and epoxide ring (1246 cm^−1^ and 915 cm^−1^) originating from DGEBA, as well as the adsorption of aliphatic ether (1113 cm^−1^) from D400, were all observed in the spectra of DDA and DDA-MMT (Figure 2c,d). In addition, a broad and strong adsorption between 3650 cm^−1^ and 3100 cm^−1^ (–OH and N–H) was firstly generated in the spectra of DDA and DDA-MMT, which originated from the nucleophilic addition reaction of amine and epoxy groups. These adsorptions indicated that the adduct and DDA-MMT were successfully synthesized, and functioned with epoxy and amine groups. Combined with the GPC results, the molecular structure of DGEBA-D400 adduct was predicted as Figure 1 (*n* = 1).

**Figure 3:**
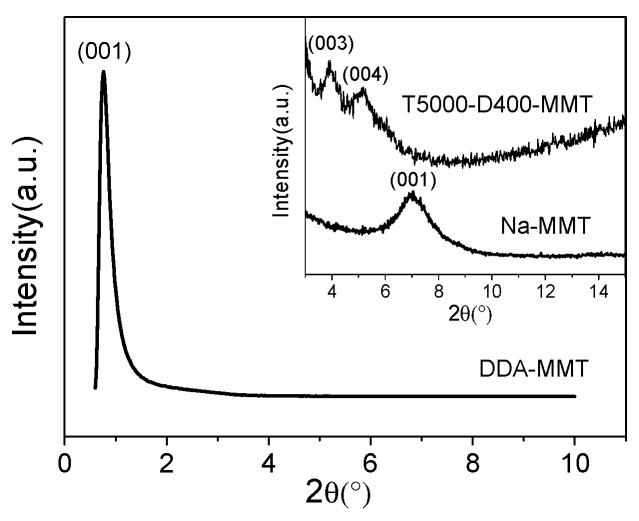
SAXS pattern of DDA-MMT and XRD patterns of T5000-D400-MMT and Na-MMT (inset).

Afterwards, SAXS pattern of DDA-MMT compared with the XRD patterns of Na-MMT and T5000-D400-MMT are presented in Figure 3. As can be observed, both DDA-MMT and Na-MMT presented a single diffraction peak at 2θ of 0.76° and 7.04°. According to Bragg’s law, the basal distances (d_(001)_) of DDA-MMT and Na-MMT were 11.61 nm and 1.25 nm, respectively. When using polyether amines T5000 and D400 as co-intercalated modifiers, the modified MMT, T5000-D400-MMT, exhibited two Bragg peaks and the d_(001)_ was 6.86 nm. Obviously, compared with Na-MMT, which was used directly without modification, both T5000-D400-MMT and DDA-MMT presented a significant increase in the basal distance, which revealed that the organic modifiers had penetrated within the interlayer of the silicate successfully via the surface functionalization.

Both results of FTIR and SAXS were consistent with our design. The DDA-MMT modified by the home-made adduct possessed the same molecular chains with the matrix on the layers surface, as well as large basal distance, resulting in low interface energy and complete compatibility with the matrix. Functionalized by the adduct terminated with epoxy and amine groups, DDA-MMT was demonstrated to be reactive to the epoxy matrix.

### 3.2. Microstructure of MMTs in Suspension

Furthermore, the microstructures of Na-MMT and DDA-MMT were investigated by SANS in detail, which is a powerful analytical method for determining micro and nano-domain structures. Due to the extremely weak absorption from nuclides in polymers, neutron scattering is a useful body probe for nanocomposite research. In addition, it could be used for the study of isotopes and magnetic materials. As a result, neutron scattering experiments can reveal more accurate and comprehensive results, which are not available from other techniques [29].

**Figure 4:**
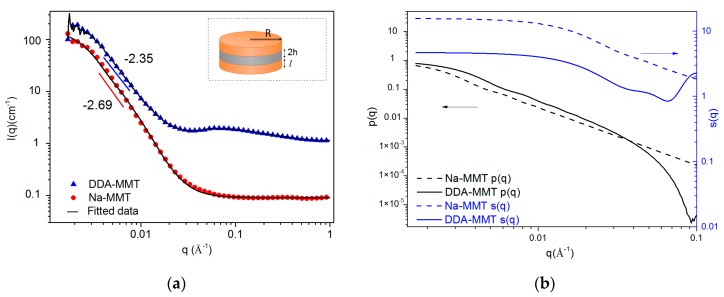
(**a**) SANS intensity profiles and fitted lines from 0.5 wt % dispersion of Na-MMT in D_2_O and DDA-MMT in ethanol according to a sandwich structure model (inset) and (**b**) the separate form and structure factors.

In this work, Na-MMT and DDA-MMT was dissolved in D_2_O and ethanol by sonicating to obtain stable suspensions with narrow distribution in particle size. The concentration of the samples was fixed at a relatively low content of 0.5 wt % to eliminate the contribution from the structure factor caused by interparticle interactions. The scattering patterns are shown in Figure 4. In this logarithmic representation, both plots of Na-MMT and DDA-MMT dispersions presented a linear region at low-q range with slopes of −2.69 and −2.35. Based on the power law, a slope of approximate −2 is predicted for cylindrical objects, and a deviation from the slope of −2 can be attributed to the influence of the structure factor, which reflects the contribution of the interaction of the scatters. Therefore, it could be speculated that more scatters of Na-MMT stacked obviously than that of DDA-MMT. 

To obtain a quantitative description of the size and aggregation of samples, a previously developed sandwich structure model was utilized, which modeled the platelet of MMT as a thin flat cylinder with the organic modifier absorbed as layers around (inset in Figure 4a). For such a model, the scattering intensity I(q) can be given by the following equation [30,35]:

(4) $$I\left(q\right)=A\phi V_{P}{\left(\rho -\rho_{m}\right)}^{2}p\left(q\right)s\left(q\right)$$

where A is an apparatus constant. ϕ, defined by ϕ = (N_p_/V_0_)V_p_, is the volume fraction of N_p_ particles with volume V_p_ each occupying suspension with volume V_0_. ρ and ρ_m_ represent the scattering length density of the particles and the matrix, which could be calculated on the basis of Equation (5):

(5) $$\rho =\frac{{\delta N}_{A}}{M}\sum b_{j}B\left(j\right)$$

where δ and M are the molecular density and the molecular mass of the scatterers, N_A_ is the Avogadro constant, b_j_ is the neutron scattering length of nucleus j, and B(j) is the number of nuclei of type j in a particle. p(q) and s(q) are the form factor and the structure factor of the scatters system, reflecting the contribution of the shape and interaction of the scatters to the scattering intensity. For a disk of radius R and height 2 h, the form factor could be expressed in Equations (6) and (7):

(6) $$p\left(q\right)=F{\left(q\right)}^{2}=4\int_{0}^{\pi /2}f^{2}\left(q,\beta \right)\sin\beta d\beta$$

(7) $$f^{2}\left(q,\beta \right)=\left(\frac{\sin^{2}\left(\mathrm{qh}\cos\beta \right)}{{\left(\mathrm{qh}\right)}^{2}\cos^{2}\beta }\right)\frac{J_{1}^{2}\left(\mathrm{qR}\sin\beta \right)}{{\left(\mathrm{qR}\right)}^{2}\sin^{2}\beta }$$

where *J*_1_ is the first integer-order Bessel function, and β is the angle between q and the normal direction of the disk.

The form factor for an organically modified clay with core thickness of 2h and surfactant layer thickness of *l* is defined as p_1_(q):

(8) $$p_{1}\left(q\right)=\int_{0}^{\pi /2}{\left[\left(\frac{\sin\left(q\left(l+h\right)\cos\beta \right)}{q\left(l+h\right)\cos\beta }\right)\times \left(\frac{\sin\left(\mathrm{qH}\cos\beta \right)}{\mathrm{qH}\cos\beta }\right)\left(\frac{2J_{1}\left(\mathrm{qR}\sin\beta \right)}{\mathrm{qR}\sin\beta }\right)\right]}^{2}\sin\beta d\beta$$

Assuming that the distance between the nearest platelets obeys a Gaussian distribution, the structure factor s(q) could be calculated based on Equation (9):

(9) $$s\left(q\right)=1+\frac{2}{N_{s}}\sum_{k=1}^{N_{s}}\left(N_{s}-k\right)\cos\left(\mathrm{kHq}\cos\beta \right)\exp\left[-k{\left(\mathrm{qH}\cos\beta \right)}^{2}\sigma_{l}^{2}/2\right]$$

where N_S_ is the number of stacked platelets in each tactoid, H represents the center-to-center distance of adjacent platelets, which equals to 2h + 2l, and $\sigma_{l}$ is the Gaussian standard deviation.

As shown in Figure 4, the SANS data in this work were fitted well with the form factor of the sandwich model and the structure factor, assuming that the nearest-neighbor distance between the platelets obeyed a Gaussian distribution. The results are presented in Table 3. The scatter length density values were obtained by Equation (5). For Na-MMT/D_2_O suspension, the layer was considered to be composed of solvent D_2_O, and the thickness was judged by half of the basal distance obtained from XRD results. The obtained radius and thickness of the MMT platelet were 925 Å and 8.3 Å, which were in good accordance with those reported in the literature [36]. Afterwards, the thickness of MMT was utilized as a fixed parameter for the fitting of all MMT dispersions. As a result, the radius and layer thickness of DDA-MMT in ethanol were 696 Å and 30.1 Å. After being functionalized by the home-made DGEBA-D400 adduct, an organic layer with a thickness of 23.8 Å (the layer thickness of DDA-MMT minus the layer thickness of Na-MMT) was introduced into interlayer. Furthermore, a significant decrease in the average number of platelets per tactoid, from 10 of Na-MMT to 3 of DDA-MMT, was also observed, which revealed that the modified MMT can be easily dispersed in organic solvent attributed to the surface functionalization, as well as the large basal distance, which could weaken the interactions between layers. Because of the decreased number in stacked layers, the overlap between layers reduced, thus a decrease in the apparent radius also observed.

### 3.3. Morphology of Nanocomposites

In order to evaluate the influence of the surface functionalization of MMT on the morphology of MMT/DGEBA nanocomposite, DDA-MMT, T5000-D400-MMT and Na-MMT were dispersed in epoxy matrix and measured by characterizations of SAXS/XRD, TEM and SANS. The content of MMT was adjusted to be 3 wt % of the total weight of the epoxy matrix.

**Figure 5:**
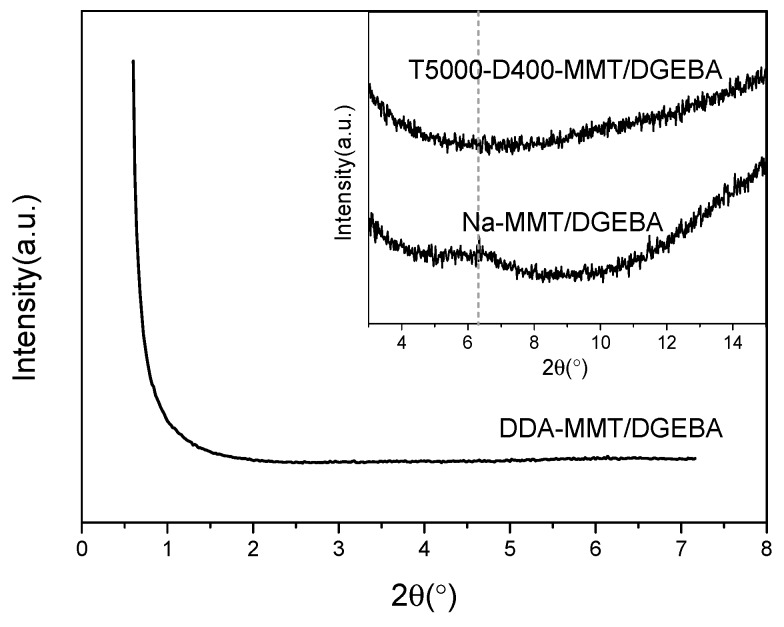
SAXS/XRD patterns of epoxy matrix nanocomposites reinforced by Na-MMT, T5000-D400-MMT and DDA-MMT at 3 wt % clay loading.

The SAXS/XRD patterns of DDA-MMT/DGEBA, T5000-D400-MMT/DGEBA and Na-MMT/DGEBA nanocomposites are presented in Figure 5. Compared to Figure 3, it can be observed that the diffraction peak position of Na-MMT changed from 2θ of 7.04° to 6.34° after being added in epoxy, with the corresponding basal spacing increasing from 1.25 nm to 1.39 nm. At the same time, the relative peak intensity also reduced. This phenomenon indicated that the basal spacing between Na-MMT platelets increased, whereas the regularity decreased during dispersion and curing processes. As for the nanocomposites reinforced by DDA-MMT and T5000-D400-MMT, the diffraction peaks of both disappeared completely, which revealed that exfoliated structures or transitional structures with various basal distances had probably been formed.

**Figure 6:**
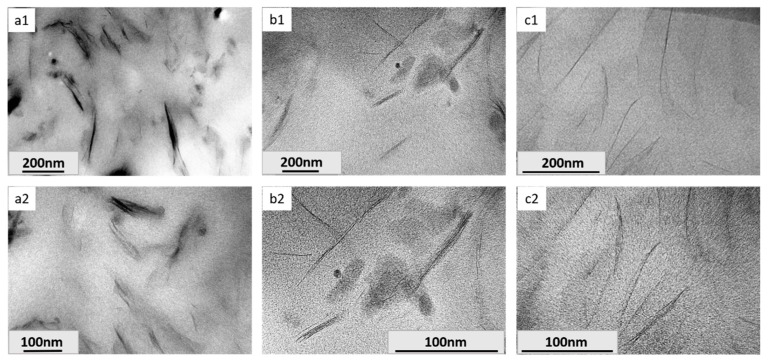
TEM images of epoxy matrix nanocomposites reinforced by Na-MMT (**a1**,**a2**), T5000-D400-MMT (**b1**,**b2**) and DDA-MMT (**c1**,**c2**) at 3 wt % clay loading.

To further investigate the fillers dispersion in detail, a TEM measurement was carried out. The micrographs are shown in Figure 6. Obviously, different kinds of morphologies were observed in the three nanocomposites. In Figure 6(a1,a2), the composite reinforced by Na-MMT exhibited as a traditional composite with two separate phases, due to the obviously agglomerated Na-MMT, which could not give full play to the advantages of nanomaterials. After surface functionalization with organic modifiers, the dispersion of fillers in both Figure 6b,c was significantly improved. From Figure 6(b1,b2), which showed the TEM images of T5000-D400-MMT/DGEBA nanocomposite, most scatterers presented typical intercalated structures with parallel orientated layers. In the current field of vision, the basal distance of the silicate layers ranged in a large field from about 30 to 60 Å, and the number of layers per tactoid was about 1 to 7. The best results were obtained in the case of epoxy nanocomposites containing clays modified by EAA (Figure 6(c1,c2)). Compared with Figure 6b1, the fillers in Figure 6c1 dispersed much more homogeneously in the matrix, and the layer number in each tactoid decreased dramatically. As can be observed at high magnification (Figure 6c2), the majority of DDA-MMT presented an exfoliated structure or a transitional structure from intercalated type to exfoliated one.

Even though the basal distance and layer number could be obtained in the TEM images, the results were limited to some local information on the surface of samples due to the small field of vision and the strong decay of electrons in polymer. Therefore, in this work, SANS measurement was performed on nanocomposites reinforced by DDA-MMT and T5000-D400-MMT to obtain a quantitative description of the size and aggregation of scatters in nanocomposites.

**Figure 7:**
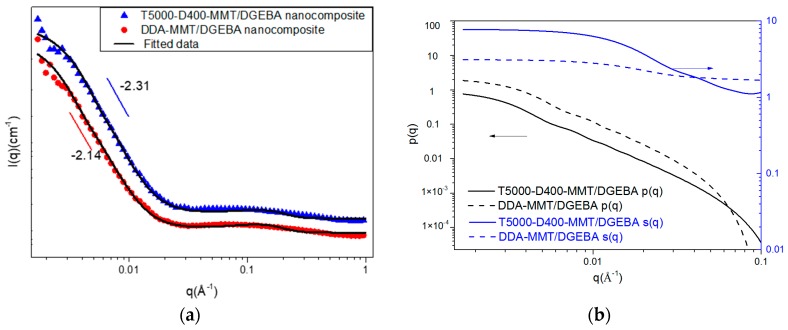
(**a**) SANS intensity profiles and fitted lines nanocomposites reinforced by DDA-MMT and T5000-D400-MMT at 3 wt % clay loading according to a sandwich structure model and (**b**) the separate form and structure factors.

As shown in Figure 7, both plots of DDA-MMT and T5000-D400-MMT reinforced epoxy presented linear regions at low-q range with slopes of −2.14 and −2.31. According to the power law mentioned in Section 3.2, the scatters in DDA-MMT/DGEBA nanocomposite had smaller size than that of the T5000-D400-MMT/DGEBA nanocomposite. Owing to the same filler loading, it can be inferred that the DDA-MMT had a better dispersion in DGEBA epoxy than that of T5000-D400-MMT. Afterwards, the scattering data were fitted based on the sandwich structure model. The fitted lines in Figure 7a followed the data well over the entire q range. The contributions of form and structure factors are shown in Figure 7b. The results calculated are presented in Table 4. The scatter length density values were obtained by Equation (5), and the volume fraction of MMT was calculated based on the weight fraction of MMT (3 wt %), the density of MMT, and the density of DGEBA/D400. As can be observed, the mean radius and layer thickness of T5000-D400-MMT in epoxy matrix were 751 Å and 19.1 Å, while for DDA-MMT, the mean radius reduced to 616 Å whereas the layer thickness increased to 30.8 Å. In addition, a significant decrease in the average number of platelets per tactoid from 5 of T5000-D400-MMT to 2 of DDA-MMT was also observed.

**Figure 8:**
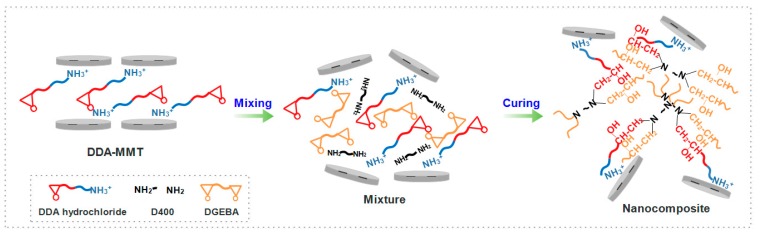
Schematic representation of the preparation process of DDA-MMT/DGEBA nanocomposite.

Based on the results obtained in SAXS/XRD, TEM and SANS, it can be inferred that compared with T5000-D400-MMT, DDA-MMT had a significant better dispersion with mostly exfoliated structure in DGEBA/D400 epoxy matrix. This could be explained by the microscopic mechanism in Figure 8. Since DDA-MMT was functionalized by DGEBA-D400 adduct which was synthesized by the two components of DGEBA/D400 matrix, it had much better compatibility and lower interface energy with the DGEBA/D400 matrix. In addition, the large basal distance obtained by SAXS was beneficial to the diffusion of epoxy monomer and curing agent into the interlayer. Considering each adduct chain was terminated with an epoxide group as well as an amine group (Figure 1), the amine group had connected with the layers via the cation exchange method, while the epoxy group would participate into the curing reactions within the interlayers. Attributed to the catalytic effect of amine cation and the higher polymerization degree of intercalation agent, the curing rate in interlayer was faster than that out of layer. Owing to the synergistic effects of molecular chain growth and curing heat release, the lamella in tactoids exfoliated successively.

### 3.4. Thermal Properties of Nanocomposites

**Figure 9:**
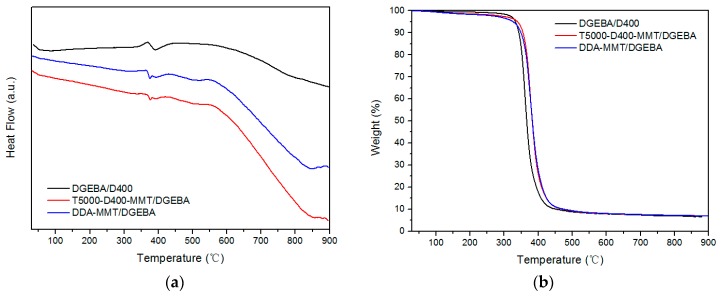
DSC (**a**) and TG (**b**) curves of neat DGEBA/D400, T5000-D400-MMT/DGEBA nanocomposite and DDA-MMT/DGEBA nanocomposites.

The DSC and TG curves of neat DGEBA/D400 epoxy resin and the nanocomposites reinforced by T5000-D400-MMT and DDA-MMT are shown in Figure 9. As can be observed, the TG curves of the nanocomposites in Figure 9b show a slight weight loss, which was probably caused by the loss of the modifier of MMT. Due to the low content of the modified MMT (3 wt %) in the nanocomposites, no obvious endothermic peak was observed in the DSC curves. In addition, a visible and wide endothermic peak appeared with a significant weight loss at 300–450 °C, which was caused by the decomposition of the epoxy resin. The decomposition temperatures for DGEBA/D400, T5000-D400-MMT/DGEBA nanocomposite and DDA-MMT/DGEBA nanocomposites are presented in Table 5. T_onset_ and T_max_ are the onset and max weight loss temperatures. T_0.1_, T_0.5_ and T_0.7_ are the temperatures at which the weight losses were 10%, 50% and 70%. Obviously, the thermal stability of the nanocomposites is better than that of the neat epoxy. When filled with 3 wt % DDA-MMT, the T_max_ of DDA-MMT/DGEBA nanocomposite was 21 °C higher than that of pure DGEBA/D400.

This may be explained by the fact that the layers of MMT suppressed the emanation of the small molecules effectively during the degradation reaction; thus, the degradation velocity of the composites was decreased. Furthermore, the strong interfacial interaction between DDA-MMT and epoxy matrix confined the activity of the polymer chains, resulting in improved heat resistance in the nanocomposite.

### 3.5. Gas Permeability of Nanocomposites

**Figure 10:**
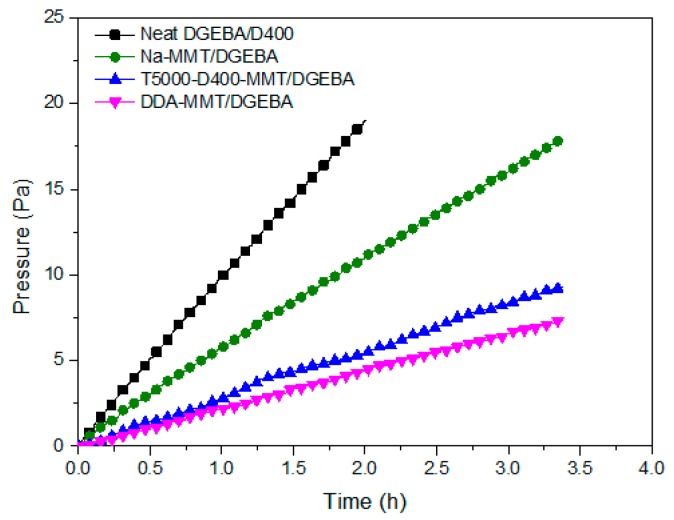
Pressure of permeated N_2_ as a function of time through neat DGEBA/D400 epoxy resin and the nanocomposites reinforced by Na-MMT, T5000-D400-MMT and DDA-MMT at 3 wt % clay loading.

As is well known, layered nanomaterials have been widely utilized as functional fillers to fabricate gas barrier nanocomposite [9,10,11,12,13,14]. In the present case, the gas permeability test was conducted on neat DGEBA/D400 epoxy resin, Na-MMT/DGEBA composite, T5000-D400-MMT/DGEBA nanocomposite and DDA-MMT/DGEBA nanocomposite respectively. The corresponding clay loading was 3 wt %. The typical curves of the permeated N_2_ pressure versus time of each sample are depicted in Figure 10. It was found that the pressure showed an increasing trend with time prolongation in all samples. With the addition of Na-MMT, T5000-D400-MMT and DDA-MMT, the increased rate of pressure decreased successively.

Based on Equations (2) and (3), N_2_ transmission rates (Q_g_, cm^3^/m^2^·24 h·Pa) and corresponding transmission coefficients (p_g_, cm^3^·cm/cm^2^·s·Pa) were calculated and presented in Table 6. Each value was an average of at least three samples. As can be seen, the N_2_ transmission coefficient of nanocomposites modified by T5000-D400-MMT and DDA-MMT were reduced by 64% and 80%, but only a slight decrease of 10% was obtained in the composite reinforced by Na-MMT. The DDA-MMT/DGEBA nanocomposite exhibited optimal N_2_ barrier performance, which was about 5 times the value of neat epoxy resin. Since the improvement in gas barrier properties of epoxy nanocomposite is strongly dependent on the dispersion/exfoliation of the layered fillers in the polymer matrix, this phenomenon may be attributable to the different morphology of the nanocomposites. As observed in Section 3.3, aggregations of layers were visible when the filler was Na-MMT. As for T5000-D400-MMT, more layers of MMT stacked together in the epoxy matrix than in the DDA-MMT; therefore, it may be concluded that the barrier effect was not so effective as that of DDA-MMT.

According to results and analysis, the barrier mechanism of MMT in epoxy composites could be proposed by the diffusion solution model, where the permeability coefficient (P) of composite can be expressed as a product of the diffusion coefficient (D) and solubility coefficient (S). With the incorporation of the impermeable layers of MMT with a volume fraction of φ, the penetrant pathways of gas molecules would become more tortuous, resulting in a prolongation of the diffusion time throughout the polymer. The permeability coefficient (P) of the nanocomposite can be expressed in Equation (10), where P_0_ is the permeability coefficient of epoxy resin. Herein, a parameter named tortuosity factor (τ) is introduced, which is defined as the ratio of the gas pathway distance in nanocomposite and polymer [37]. Therefore, when at a certain filler ratio, it can be inferred that the gas barrier property of composite is mainly influenced by the tortuosity factor, which related to the fillers aspect ratio, dispersion, exfoliation, and orientation.

(10) $$\frac{P}{P_{0}}=\frac{1-\varphi }{\tau }$$

On the basis of the nanocomposites morphology obtained by XRD, TEM and SANS, the lamellae of the modified MMT in the present study are randomly dispersed, whereas the stacked-structures still exist. Therefore, both the orientation and stacking of the lamellae should be considered for the calculation of the tortuous radius.

**Figure 11:**
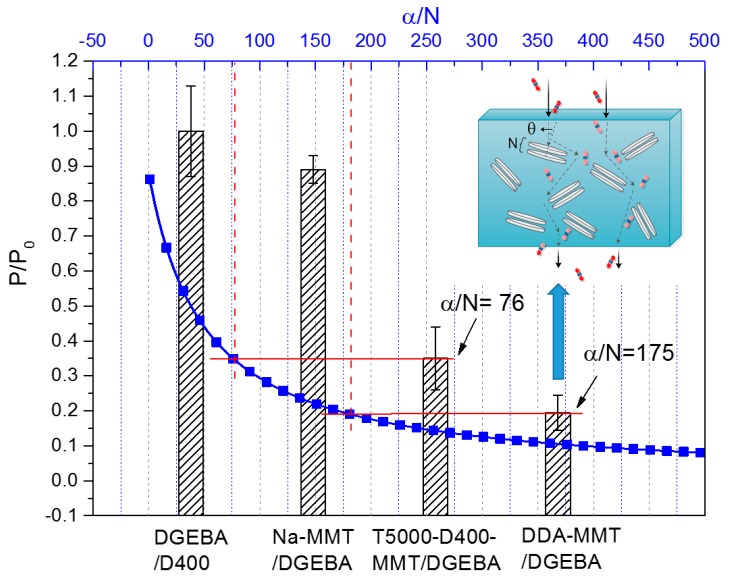
P/P_0_ of neat DGEBA/D400 epoxy resin and the corresponding nanocomposites reinforced by Na-MMT, T5000-D400-MMT and DDA-MMT respectively at 3 wt % clay loading. The dotted line shows the prediction curve of P/P_0_ as a function of the aspect ratio (α)/number of layers (N) of fillers, according to the modified Nielsen model. (The density of Na-MMT is 2.66 g·cm^−3^ [35] and the density of DGEBA/D400 resin was measured as 1.08 g·cm^−3^.).

To evaluate the effect of the orientation of the MMT layers, an order parameter S’ is introduced to represent the orientation of the nano-layers:

(11) $$S^{\prime }=\frac{3\langle \cos^{2}\theta \rangle -1}{2}$$

where θ is the angle between the direction of gas diffusion and the normal direction of the lamella, as shown in Figure 11 (inset). The angular brackets denote averaging over all the sheets. For layers with perfect alignment, S’ = 1 (θ = 0), while for layers with random distribution, S’ = 0 [13,38,39].

According to the modified Nielsen model [13], the tortuosity factor can be determined as Equation (12), where α is the aspect ratio of the lamella.

(12) $$\tau =1+\frac{\alpha }{3}\left(S^{\prime }+\frac{1}{2}\right)\varphi$$

Taking into consideration the nanocomposites at high filler loadings, the tortuosity factor should be modified based on the number of the layers (N) in each tactoid, which would decrease the apparent aspect ratio into α/N. For the present study, the platelets are randomly dispersed but the stacked-structure may still exist. Thus, a combination of Equations (10) and (12) is used for modeling, as Equation (13) shows:

(13) $$\frac{P}{P_{0}}=\frac{1-\varphi }{1+\frac{\alpha }{3N}\left(S^{\prime }+\frac{1}{2}\right)\varphi }$$

As can be observed, there are two key factors in this process, including the filler properties (e.g., aspect ratio and loading) and the extent of dispersion and exfoliation. In addition, strong interfacial interactions between inorganic fillers and organic matrix could prevent the generation of imperfections.

For the present study, values of P/P_0_ of pristine epoxy and MMT modified composites are shown in Figure 11. The dotted line was the predicted value of P/P_0_ as a function of α/N of fillers, based on Equation (13) by using 0 for the order parameter S’, which meant that the fillers dispersed randomly throughout the matrix. From Figure 11, it may be clearly seen that the values of P/P_0_ were substantially decreased with the increase of α/N value. As a result, the α/N value of nanocomposite reinforced by DDA-MMT and T5000-D400-MMT was 175 and 76. Since the aspect ratio of raw Na-MMT was about 300–400, it can be inferred that the average number of layers in each tactoid was about 2 in the DDA-MMT/DGEBA nanocomposite, and 4 or 5 in the T5000-D400-MMT/DGEBA nanocomposite. This was in good accordance with the SANS results, indicating that the DDA-MMT was almost exfoliated and homogeneously dispersed in the nanocomposite.

## 4. Conclusions

A new kind of modifier DGEBA-D400 adduct (DDA) for layered nanomaterial (MMT) was synthesized by reacting epoxy monomer DGEBA with curing agent D400, which could provide not only complete compatibility, but also reactivity with DGEBA/D400 epoxy matrix, resulting in better dispersion, as well as strong interface interaction. By manufacturing DGEBA matrix nanocomposites reinforced by Na-MMT, DDA-MMT and T5000-D400-MMT, the effects of the surface functionalization of MMT on the nanocomposites’ morphology and gas barrier property were systematically investigated. Based on XRD, TEM, and SANS results, the optimal dispersion/exfoliation was observed in the DDA-MMT/DGEBA nanocomposite, with a mean radius of 751 Å, a layer thickness of 30.8 Å, and only two stacked layers in each tactoid attributed to the compatible and reactive modifier of DDA. Furthermore, the DDA-MMT/DGEBA nanocomposite showed the best N_2_ barrier performance. At 3 wt % clay loading, the N_2_ barrier property of DDA-MMT/DGEBA nanocomposite was about 5 times the value of neat epoxy resin. Based on a modified Nielsen model, it was clarified that the improvement in gas barrier property was due to the evenly dispersed lamellas with almost exfoliated structures. The average number of layers in each tactoid was about 2 in the DDA-MMT/DGEBA nanocomposite, which was consistent with the SANS results. These extraordinary characteristics confirmed the superiority of the epoxy-diamine adduct, which will effectively improve the gas barrier property of layered silicate/epoxy nanocomposites.

## Figures and Tables

**Table 1:** Preparation methods for DGEBA/D400 resin and MMT/DGEBA nanocomposites at 3 wt % clay loading.

Sample	Modified MMT	Intercalating Sequence		Mixing Method
		First Modifier	Second Modifier	
DGEBA/D400	——	——	——	Mechanical agitating for 30 h
Composite 1	Na-MMT	——	——	Mechanical agitating for 30 h and ball-milling for 60 h
Composite 2	T5000-D400-MMT	D400	T5000	
Composite 3	DDA-MMT	DGEBA-D400 Adduct	——	

**Table 2:** Molecular weights and distribution of DGEBA-D400 adduct.

Peak	M_n_ (g/mol)	M_w_ (g/mol)	M_w_/M_n_	Peak Area (%)
1	1460	2022	1.4	65
2	368	407	1.1	35

**Table 3:** Fitting results obtained from SANS for 0.5 wt % dispersions of Na-MMT and DDA-MMT ^1^.

Sample	Radius (Å)	Platelet Thickness (Å)	Layer Thickness (Å)	No. of Platelets per Tactoid
Na-MMT/D_2_O	925 ± 22	8.3 ± 0.1	6.3 ± 0.2 ^2^	10 ± 1
DDA-MMT/Ethanol	696 ± 20	8.3 ± 0.1 ^3^	30.1 ± 0.3	3 ± 2

^1^ Fixed parameters were $\rho_{\mathrm{Na}-\mathrm{MMT}}=3.860\times 10^{-6}{Å}^{-2}$, $\rho_{D_{2}O}=6.370\times 10^{-6}{Å}^{-2}$, $\rho_{\mathrm{DDA}}=0.785\times 10^{-6}{Å}^{-2}$, $\rho_{\mathrm{ethanol}}=-0.345\times 10^{-6}{Å}^{-2}$. The errors reported were the statistical uncertainties on each parameter, which were calculated during the fitting process; ^2^ Obtained from the XRD results; ^3^ Fixed parameters obtained from results of Na-MMT/D_2_O suspension.

**Table 4:** Fitting results obtained from SANS for nanocomposites reinforced by DDA-MMT and T5000-D400-MMT at 3 wt % filler loading ^1^.

Sample	Radius (Å)	Platelet Thickness (Å)	Layer Thickness (Å)	No. of Platelets per Tactoid
T5000-D400-MMT/DGEBA nanocomposite	751 ± 11	8.3 ± 0.1 ^2^	19.1 ± 0.1	5 ± 1
DDA-MMT/DGEBA nanocomposite	616 ± 8	8.3 ± 0.1 ^2^	30.8 ± 0.2	2 ± 1

^1^ Fixed parameters were φ = 0.12, $\rho_{\mathrm{Na}-\mathrm{MMT}}=3.860\times 10^{-6}\;{Å}^{-2}$, $\rho_{\mathrm{DGEBA}}=1.530\times 10^{-6}\;{Å}^{-2}$, $\rho_{\mathrm{DDA}}=0.785\times 10^{-6}\;{Å}^{-2}$, $\rho_{T5000}=0.339\times 10^{-6}\;{Å}^{-2}$, $\rho_{D400}=0.292\times 10^{-6}\;{Å}^{-2}$. The errors reported were the statistical uncertainties on each parameter, which were calculated during the fitting process; ^2^ Fixed parameters obtained from the results of Na-MMT.

**Table 5:** Decomposition temperatures for DGEBA/D400, T5000-D400-MMT/DGEBA nanocomposite and DDA-MMT/DGEBA nanocomposites.

Sample	T_onset_ (°C)	T_0.1_ (°C)	T_0.5_ (°C)	T_0.7_ (°C)	T_max_ (°C)
**DGEBA/D400**	308	343	366	379	360
**T5000-D400-MMT/DGEBA**	327	355	382	395	377
**DDA-MMT/DGEBA**	321	349	383	397	381

**Table 6:** N_2_ transmission rate and coefficient of neat DGEBA/D400 epoxy resin and MMT/DGEBA nanocomposites.

Samples	DGEBA/D400	Na-MMT/DGEBA	T5000-D400-MMT/DGEBA	DDA-MMT/DGEBA
N_2_ transmission rate ($Q_{g}$, ×10^−5^ ${\mathrm{cm}}^{3}/m^{2}\cdot 24h\cdot \mathrm{Pa}$)	4.4 ± 0.7	4.3 ± 0.3	1.7 ± 0.5	1.1 ± 0.3
N_2_ transmission coefficient ($p_{g}$, ×10^−15^ ${\mathrm{cm}}^{3}\mathrm{cm}/{\mathrm{cm}}^{2}\cdot s\cdot \mathrm{Pa}$)	5.9 ± 0.8	5.3 ± 0.3	2.1 ± 0.6	1.2 ± 0.4
